# Circuit Models and Experimental Noise Measurements of Micropipette Amplifiers for Extracellular Neural Recordings from Live Animals

**DOI:** 10.1155/2014/135026

**Published:** 2014-07-16

**Authors:** Chang Hao Chen, Sio Hang Pun, Peng Un Mak, Mang I Vai, Achim Klug, Tim C. Lei

**Affiliations:** ^1^State Key Laboratory of Analog and Mixed-Signal VLSI, University of Macau, Taipa 999078, Macau; ^2^Department of Electrical and Computer Engineering, Faculty of Science and Technology, University of Macau, Taipa 999078, Macau; ^3^Department of Physiology and Biophysics, University of Colorado School of Medicine, Aurora, CO 80045, USA; ^4^Department of Electrical Engineering, University of Colorado Denver, Denver, CO 80217-3364, USA

## Abstract

Glass micropipettes are widely used to record neural activity from single neurons or clusters of neurons extracellularly in live animals. However, to date, there has been no comprehensive study of noise in extracellular recordings with glass micropipettes. The purpose of this work was to assess various noise sources that affect extracellular recordings and to create model systems in which novel micropipette neural amplifier designs can be tested. An equivalent circuit of the glass micropipette and the noise model of this circuit, which accurately describe the various noise sources involved in extracellular recordings, have been developed. Measurement schemes using dead brain tissue as well as extracellular recordings from neurons in the inferior colliculus, an auditory brain nucleus of an anesthetized gerbil, were used to characterize noise performance and amplification efficacy of the proposed micropipette neural amplifier. According to our model, the major noise sources which influence the signal to noise ratio are the intrinsic noise of the neural amplifier and the thermal noise from distributed pipette resistance. These two types of noise were calculated and measured and were shown to be the dominating sources of background noise for *in vivo* experiments.

## 1. Introduction

Neurons in the brain communicate via action potentials, which are small and fast changes in the voltage of the cell membrane [1]. During periods of inactivity, the cell membrane of a neuron is typically hyperpolarized to about −60 mV (cell's interior environment negative). During periods of activity, the membrane potential depolarizes and subsequently repolarizes over a period of about one to several milliseconds [1]. Action potential is the unit of information processing in neurons, and as a result many neuroscience research projects involve recordings of action potentials or action potential sequences from single neurons or neural networks. One way to record action potentials is to use high-impedance extracellular electrodes that are advanced into brain tissue and placed directly next to a single neuron, allowing for the extracellular recording of action potentials through the electrode [2–6]. The signal is sent to an amplifier, digitized, and subsequently evaluated [7]. For extracellular recording, the action potential voltage can be as low as just a few microvolts, making it challenging to record it reliably against various sources of noise. Alternatively, an investigator may attempt to impale the neuron of interest with the electrode or to establish an electrical connection to the neuron's interior via a patch clamp. While these techniques result in larger signals which are easier to measure, intracellular or patch-clamp techniques are very challenging in live animals, making extracellular recordings the technique of choice for many investigators.

Voltage spikes acquired in extracellular recording are typically between 50 *μ*V to 500 *μ*V peak-to peak, with rise times of 0.2 ms or more and pulse durations of 1 ms or more [8]. Amplification is required before these small signals can be analyzed. In addition, several intrinsic and extrinsic noise sources are present during the recordings, affecting the signal-to-noise ratio of the measured voltages. Thus, it is not only important to use low-noise recording amplifiers, but also an understanding of these noise sources is required such that a strategy to eliminate or to minimize them can be developed. Several studies [8–18] have been published describing designs of low noise neural acquisition amplifiers, and a variety of commercially available amplifiers currently exist in the market and are used by neuroscientists. However, most of these studies only discuss how to reduce the intrinsic noise of the amplifier. Discussions of various biological noise sources as well as the electrode noise are largely lacking. For example, the role of the electrode's input impedance, which is one of the important parameters for extracellular recordings, has not received much attention in many designs [10–12, 14–18]. Choosing electrodes with suitable impedances makes the amplifier design appropriate for recording local field potential, which results from the activity of small neural networks, or appropriate for recording activity from a single neuron extracellularly.

For* in vivo* recordings from neurons, both Yang et al. [19] and Lopez et al. [20] have proposed noise models to study the multiple noise sources that need to be considered for the recording. However, these models are largely based on the use of metal electrodes, such as tungsten, platinum, or titanium nitride electrodes, not glass micropipettes. Metal electrodes generally have better noise performance than glass micropipette electrodes [13, 19, 20]. For example, Millar and Barnett [13] reported that there is 65 *μ*V peak-to-peak thermal noise generated from a 1 MΩ glass micropipette over a recording frequency range between 100 Hz to 8 kHz, while a tungsten electrode in the same condition usually shows a noise level of ~20–50 *μ*V peak-to-peak. The reason that noise generated from a metal electrode is less than that of a glass micropipette is because the impedance of a metal electrode is largely capacitive [21], resulting in a smaller real component (resistance) to generate thermal noise. While these types of metal electrodes are widely used, they have some disadvantages. For example, metal electrodes cannot easily be combined with microiontophoretic drug testing [22–45]. Also, glass pipettes are typically produced by the investigator with programmable electrode pullers that allow for the adjustment of many different parameters, resulting in virtually endless possibilities in adjusting the shape of the electrode tip [4, 46]. Finally, only glass pipettes can be filled with dyes, viral constructs, tracers, and other materials that can be ejected during the experiment for various purposes [47, 48]. One example is that we have developed a piggyback multibarrel glass pipette system to measure neural signals and to simultaneously inject chemical agents to manipulate neural responses [23]. It is therefore not surprising that both glass and metal electrodes are widely used.

A number of noise models have been proposed for various forms of neural recording [8–16, 18–20, 49]. Yet there is still a lack of a comprehensive noise model to describe extracellular neural recording using glass micropipettes as the recording electrode. For example, most of the studies [10–12, 14–18] only described intrinsic noise generated by the amplifiers themselves. Chae et al. [9], Budai [8] and Millar and Barnett [13] discussed the additional noise of power-line interference but noise of biological origins and noise generated from electrodes were not included in their models. Yang et al. [19] and Lopez et al. [20] included both biological and electrode noise but their models are for metal electrodes only. When using glass micropipettes as recording electrodes, other noise sources, such as dielectric noise generated from the distributed capacitance of the glass pipette wall [49, 50], have to be considered. Reference [49] modeled the glass micropipette which has a membrane-to-glass seal to describe the situation of intracellular recording inside the cell membrane of a neuron. In extracellular recording single cells selectively are determined by the position of the micropipette tip relative to the cell membrane. Additional noise will arise from nearby cells and from the cerebrospinal fluids. Therefore, a comprehensive mathematical model to describe all noise generated during extracellular recording using glass microelectrodes is necessary and is proposed in this paper.

The purpose of this study is to propose an equivalent circuit model and a noise model for* in vivo* extracellular neural recording using glass micropipettes and to test models using a two-stage amplifier design. Experiments using dead brain tissue from a gerbil and also directly measuring neural activities triggered by external auditory stimulations from the brain of an anesthetized gerbil were performed to further verify our models. According to our simulation and experimental results, the major noise sources which influence the signal-to-noise ratio (SNR) are the intrinsic noise of neural amplifier and the thermal noise from distributed pipette resistance.

## 2. Methods

The purpose of this study was to assess the various noise sources that affect extracellular recordings and to create model systems in which novel amplifier designs could be tested. In the first part of this manuscript, we develop an equivalent mathematical model that accurately describes the various noise sources involved in extracellular recordings with glass micropipettes. In the second part, we describe several measurement schemes to measure noise performance and the amplification efficacy of the proposed micropipette neural amplifier.

### 2.1. Circuit Modeling for Micropipette Neural Amplifier

#### 2.1.1. Equivalent Circuit Model of a Micropipette for Extracellular Neural Recording

**Figure 1:**
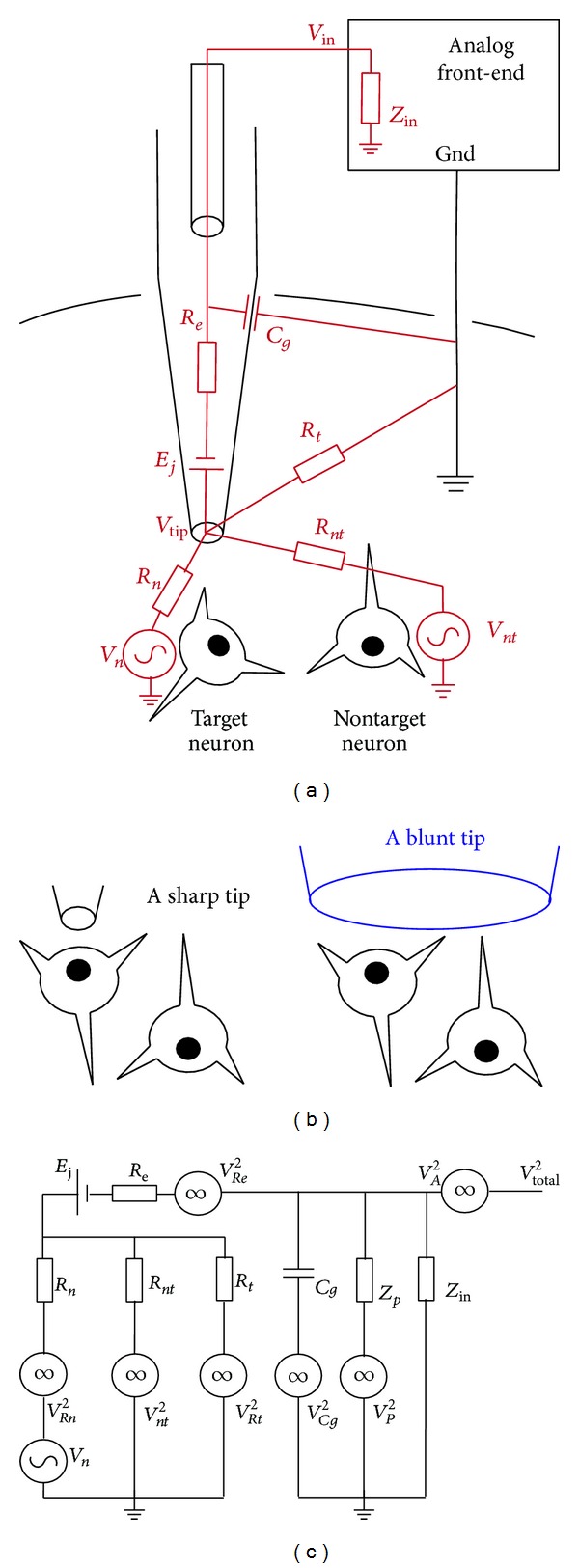
(a) Equivalent circuit model of a micropipette used for* in vivo* extracellular neural recordings of conscious animals. *V*_*n*_: neural voltage of target neuron; *V*_*nt*_: neural voltage of non-target neurons; *R*_*n*_: equivalent resistance between target neuron and the micropipette tip; *R*_*nt*_: equivalent resistance between nontarget neurons and the micropipette tip; *R*_*t*_: equivalent resistance between the micropipette tip and the ground; *V*_tip_: micropipette tip voltage; *E*_*j*_: liquid junction potential and half-cell potential; *R*_*e*_: distributed resistance of the electrolyte; *C*_*g*_: equivalent capacitance of the glass pipette wall; *Z*_in_: overall input impedance of the amplifier; *V*_in_: input voltage of the amplifier. (b) Illustrations of using a sharp micropipette and a blunt micropipette. (c) Equivalent noise model of a glass micropipette connected to a neural amplifier. (*V*_*Rn*_^2^: thermal noise generated from equivalent resistor between target neuron and the micropipette tip; *V*_*nt*_^2^: noise generated from nontarget neurons; *V*_*Rt*_^2^: thermal noise generated from equivalent resistor between the micropipette tip and the ground; *V*_*Cg*_^2^: dielectric noise of the micropipette wall; *V*_*p*_^2^: environmental noise; *V*_*Re*_^2^: thermal noise generated by the distributed resistance of the electrolyte; *V*_*A*_^2^: intrinsic noise of the neural amplifier; *Z*_*p*_: equivalent impedance between the amplifier and the power line. *V*_total_^2^: total noise of the micropipette neural amplifier).

Figure 1(a) is the equivalent circuit model of a glass micropipette connected to the front-end of a neural signal amplifier for* in vivo* extracellular neural recording in a live rodent. This model is based on previous studies which examine the various physical phenomena attributed to noise generated in extracellular recording [8, 13, 19, 20, 49–56]. Our goal is to provide a unified yet simple mathematical model to help understand the important noise factors for neural recordings. For these types of* in vivo* recordings, the micropipette tip is typically positioned outside of but very close to the cell membrane. As shown in our equivalent circuit model, *V*_*n*_ *and V*_*nt*_ represent the extracellular neural voltages generated by a target neuron which one would like to record and other nontarget neurons surrounding the target neuron, respectively. *R*_*n*_ *and R*_*nt*_ are the equivalent resistance which span between the respective neurons (target and nontarget) and the glass micropipette tip. The resistance of *R*_*n*_ *and* the resistance of *R*_*nt*_ are proportional to both the electrical resistivity of the cerebrospinal fluid and the distance between the membrane of the respective cells and the micropipette tip. In addition, the resistive values of *R*_*n*_ *and R*_*nt*_ largely depend on the physical locations of the neurons against the micropipette tip. If the opening of the micropipette tip is directly above the target neuron, the majority of the ionic current induced at the neuron membrane can be captured by the micropipette tip; hence, the resistance *R*_*n*_ is significantly reduced. On the other hand for the surrounding nontarget neurons, the ionic currents generated by these neurons will have difficult time getting inside the micropipette and instead will be dispersed in the cerebrospinal fluid and eventually captured by the ground, resulting in a significant increase of the *R*_*nt*_ resistance to the micropipette tip. For this reason, *R*_*nt*_ is assumed to be significantly larger than *R*_*n*_  (*R*_*nt*_ ≫ *R*_*n*_). *R*_*t*_ is an equivalent resistor representing the electrical resistance between the ground and the micropipette tip. *R*_*t*_ is also proportional to both the cerebrospinal fluid resistivity and the distance between the micropipette tip and the ground.

*V*_tip_ is the voltage at the opening of the micropipette tip and can be estimated based on the neural voltages and the equivalent resistances of cerebrospinal fluid,

(1) Vtip=VnRnt||RtRn+Rnt||Rt+VntRn||RtRnt+Rn||Rt=Vn1Rn/(Rnt||Rt)+1+Vnt1Rnt/(Rn||Rt)+1.

One method of understanding how neurons of a specific brain area process information involves measurements of neural voltages generated from single neurons. To do this, the pipette tip is placed very close to the target neuron, resulting in the resistive value of *R*_*n*_ significantly smaller than those of *R*_*nt*_ and *R*_*t*_. Thus, *R*_*n*_/(*R*_*nt*_||*R*_*t*_) ≈ 0 and *R*_*nt*_/(*R*_*n*_||*R*_*t*_) ≫ 1, and (1) reduces to

(2) $$\begin{array}{c} V_{\text{tip}}\approx V_{n}. \end{array}$$

If a blunt micropipette tip is used for* in vivo* extracellular recording, several neurons can be simultaneously located under the tip of the micropipette (*R*_*n*_ ≈ *R*_*nt*_), as shown in Figure 1(b). In this case, *V*_tip_ will pick up voltages from multiple neurons, which is not desirable for understanding specific neural function.

The inside of the micropipette can be further modeled. A liquid junction potential is formed inside the pipette due to the ionic concentration difference between the electrolyte in the glass micropipette and the cerebrospinal fluid. In addition, a half-cell potential is also present due to the electrode-electrolyte interface. These two potentials can be modeled together by a voltage source (*E*_*j*_) in our equivalent circuit model. *R*_*e*_ is the distributed resistance of the electrolyte filled inside the micropipette. *C*_*g*_ is the equivalent capacitor between the electrolyte and the electrical ground located in the cerebrospinal fluid separated by the glass wall of the micropipette. Finally, *Z*_in_ and *V*_in_ are the input impedance and the input voltage of the analog amplifier, respectively. Therefore, *V*_in_ can be modeled based on the equivalent circuit model of the micropipette as follows:

(3) $$\begin{array}{c} V_{\text{in}}\approx \left(V_{\text{tip}}+E_{j}\right)\frac{Z_{Cg}||Z_{\text{in}}}{R_{e}+Z_{Cg}||Z_{\text{in}}}, \end{array}$$

where *Z*_*Cg*_ = 1/*j*2*πfC*_*g*_ and *f* is the working frequency. For a high quality glass micropipette, *C*_*g*_ is typically less than 0.1 pF [49, 50] and the input impedance of the analog front-end *Z*_in_ is typically two orders of magnitude larger than *Z*_*Cg*_. Using a sharp micropipette to record single cell activity (*V*_tip_ ≈ *V*_*n*_), (3) is further simplified to

(4) $$\begin{array}{c} V_{\text{in}}\approx \left(V_{n}+E_{j}\right)\frac{Z_{\text{in}}}{R_{e}+Z_{\text{in}}}. \end{array}$$

Equation (4) gives us two important circuit design guidelines for* in vivo* extracellular neural recording. In order to optimally measure the neural voltage generated by a single neuron (*V*_in_ ≈ *V*_*n*_), first the DC offset *E*_*j*_ should be rejected; otherwise, it will be amplified together with the neural voltage and may saturate the subsequent stages of amplification. Second, the input impedance of the analog front-end (*Z*_in_) should be significantly larger than the resistance of the electrolyte (*R*_*e*_) to avoid signal reduction at the input front-end.

#### 2.1.2. Noise Analysis of the Micropipette Neural Amplifier

Figure 1(c) shows the electronic noise model [19, 20, 49, 51, 53, 57–60] for our equivalent circuit model for* in vivo* micropipette recordings. *V*_*Rn*_^2^ is the thermal noise generated from the equivalent resistance between the target neuron and the micropipette tip. Thermal noise can be estimated based on the well-known Johnson-Nyquist thermal noise relation [51, 54, 61]

(5) $$\begin{array}{c} V_{R}^{2}=4k_{B}TR, \end{array}$$

where *k*_*B*_ is the Boltzmann's constant, *T* is the temperature in Kelvin, and *R* is the equivalent resistance. For single cell recording where the tip is very close to the target neuron, *V*_*Rn*_^2^ can be simply neglected due to the small resistance. *V*_*nt*_^2^ is the noise generated from other nontarget neurons. As explained above, voltages from nontarget neurons registered at the micropipette tip are small when a sharp tip is used. However, the voltages generated from nontarget neurons can still contribute to the background noise [19, 52, 55, 62–64] superimposed on the target neural signal. This background noise can be determined by using

(6) $$\begin{array}{c} V_{nt}\left(t\right)=\overset{}{\underset{i}{\sum }}\overset{}{\underset{k}{\sum }}V_{nt,i}\left(t-t_{i,k}\right); \end{array}$$

*V*_*nt*_(*t*) is the algebraic sum (background noise) of all nontarget neural voltages. *V*_*nt*,*i*_(*t* − *t*_*i*,*k*_) is the neural voltage at the micropipette tip of a neuron *i* firing a sequence of neural action potentials at various time instants *t*_*i*,*k*_. This overall background noise *V*_*nt*_(*t*) contributes a 1/*f* ^*x*^ noise spectrum (*x* ≈ 0.5 to 1.5) in the frequency domain [19, 52, 55]. Because of the 1/*f* ^*x*^ nature of this background noise which dominates in the low frequencies [19, 52, 55, 58, 63], it can be rejected using a high-pass filter after the unity-gain first stage amplification. *V*_*Rt*_^2^ is the thermal noise generated by the equivalent resistance between the ground and the micropipette tip. This thermal noise can also be neglected due to the small tip opening in consideration.

There are also noise sources inside the micropipette. *V*_*Re*_^2^ is the thermal noise generated from distributed resistance of the electrolyte and can be estimated using (5). This thermal noise can be minimized by reducing the distributed resistance by increasing the ion concentration of the electrolyte inside the micropipette. *V*_*Cg*_^2^ represents the dielectric noise generated from the distributed pipette capacitance of the glass pipette wall. This noise can be described using the following equation [49, 50]:

(7) $$\begin{array}{c} V_{cg}^{2}\left(f\right)=4kTDC_{g}\left(2\pi f\right), \end{array}$$

where *D* is the dissipation factor of the glass material. Equation (7) shows that the noise generated from the pipette wall dominates in higher frequencies. For the frequencies of interest in neural recoding, this noise can be minimized using high quality glass pipette having a *D* < 0.0001.

There are also noise sources from the environment. *V*_*P*_^2^ represents all noise generated from the environment, in particular the 50/60 Hz power line noise. *Z*_*p*_ is the equivalent impedance between the amplifier input and power line. For this reason, the amplifier front-end should be carefully designed to eliminate any electronic ground loops [8, 13] to avoid coupling environmental noise to the amplifier. Thus, in our model the impedance of *Z*_*p*_ is assumed to be larger than *Z*_in_  (*Z*_*p*_ ≫ *Z*_in_). Finally,  *V*_*A*_^2^ is the intrinsic noise from the amplifier, which is inherited due to the imperfection of circuit elements in the neural amplifier. Careful design of the low-noise amplifier front-end and subsequent amplifier stages is crucial to obtain optimal neural signal.

Using the aforementioned model, assuming these noise sources are independent, the overall input referred noise of the micropipette neural recording amplifier for conscious rodents can be summarized as

(8) Vtotal2=VA2+VP2|Zin||Re||ZCgZP+Zin||Re||ZCg|2+VCg2|Zin||Re||ZPZCg+Zin||Re||ZP|2+[Ej2+VRe2+VRn2(Rt||RntRn+Rt||Rnt)2+Vnt2(Rt||RnRnt+Rt||Rn)2+VRt2(Rnt||RnRt+Rnt||Rn)2]×|Zin||ZCg||ZPRe+Zin||ZCg||ZP|2.

To simplify the above equation in order to obtain a high SNR, several considerations are necessary to prepare the micropipettes and to design the neural amplifier. First, the amplifier should be well-designed to avoid any environmental interference, such that *Z*_*p*_ ≫ *Z*_in_. Second, the glass micropipette should be made out of good quality glass such that the distributed capacitance *C*_*g*_ is small enough to make *Z*_*Cg*_ much larger than *Z*_in_. Third, the half-cell potential and the liquid junction potential (*E*_*j*_) should be rejected by using a high-pass filter after the analog front-end. It is reported that the liquid junction potential has a voltage of several millivolts to several hundred millivolts, depending on the concentration and chemical composition of the electrolytes [56, 65]. If these DC voltage offsets are not rejected, the amplifier gain may be saturated resulting in difficulty measuring the neural voltages. Finally, a fine-tip glass micropipette should be used and placed close to the target neuron. Equation (8) then reduces to

(9) $$\begin{array}{c} V_{\text{total}}^{2}\approx V_{A}^{2}+V_{Re}^{2}{\left|\frac{Z_{\text{in}}}{R_{e}+Z_{\text{in}}}\right|}^{2}. \end{array}$$

From (4), the neural voltage of the target neuron *V*_*n*,in_ presented at the analog front-end is approximately

(10) $$\begin{array}{c} V_{n,\text{in}}\approx V_{n}\frac{Z_{\text{in}}}{R_{e}+Z_{\text{in}}}. \end{array}$$

Thus, the overall input referred SNR in the bandwidth of (*f*_1_ − *f*_2_) can be estimated by

(11) SNR =|Vn|(1/|Zin/(Re+Zin)|2)∫f2f1|VA|2df+|VRe|2(f1−f2).

Equation (11) indicates that the overall input referred noise is mainly contributed by the intrinsic noise of the amplifier and the thermal noise arising from the distributed resistance of the electrolyte contained in the glass micropipette. Therefore, it is important to carefully design the amplifier to achieve low intrinsic noise, as well as adjusting the electrolyte concentration to reduce the overall noise for recording.

### 2.2. Overall Gain and Input Impedance Equations

A typical extracellular action potential is on the order of 50–500 *μ*Vpp with a frequency bandwidth of 100 Hz to 5 kHz [8]. To record such a small voltage, a low-noise high-quality analog amplifier is required. As mentioned in the previous section, the amplifier should have relatively low intrinsic noise compared to the extracellular neural voltages. Meanwhile, a high-pass filter should be introduced to reject the DC offset induced by the liquid junction at the micropipette tip and the half-cell potential at the metal-electrolyte interface. In addition, according to (11), the input impedance of the analog front-end should be designed to be as large as possible so that the overall SNR can be improved.

**Figure 2:**
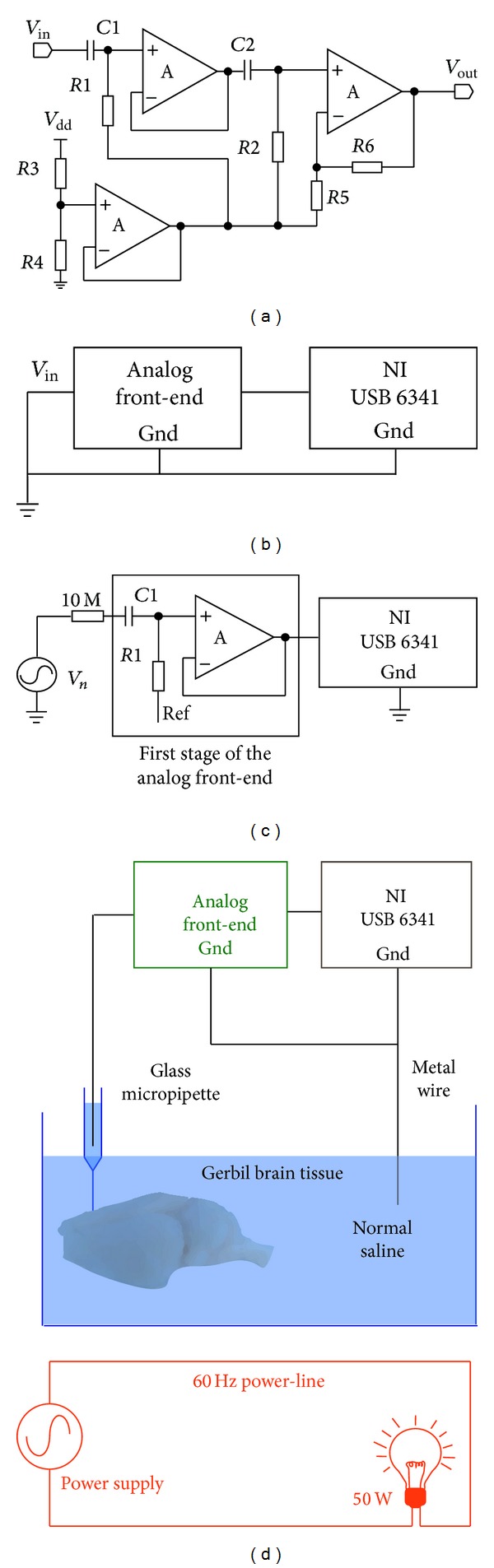
(a) Circuit diagram of the micropipette neural amplifier deigned for* in vivo* single-cell extracellular neural recordings of conscious rodents using a sharp-tipped glass micropipette. (*C*1 = 1.5 nF, *C*2 = 0.1 *μ*F, *R*1 = 1 GΩ, *R*2 = 20 KΩ, *R*3 = 27 KΩ, *R*4 = 10 KΩ, *R*5 = 1 KΩ, and *R*6 = 200 KΩ) (b) Schematic diagram illustrating the experimental setup used to measure the intrinsic input-referred noise of the neural amplifier. (c) Schematic diagram of measuring the input capacitance of the neural amplifier. (d) Experimental setup to characterize the overall noise of the neural amplifier with the glass micropipette (light off) and the environmental noise (light on) generated by a 60 Hz power-line attached to a light bulb using dead gerbil brain tissue submerged in normal saline.

Figure 2(a) shows the circuit diagram of our neural amplifier used in the measurements reported in this paper. The amplifier is designed to record extracellular neural activities of conscious rodents based on the analysis discussed in the previous section. Our neural amplifier implementation is a two-stage design to achieve a unity gain (*A*_1_ = 1) for the first stage and a gain of 200 (*A*_2_ = 200) for the second stage. The overall voltage gain (*A*_0_) of our amplifier can be mathematically expressed as

(12) A0(f)=A1(f)·A2(f)=[R1||Zin,AR1||Zin,A+(1/j2πfC1)]·[R2||Zin,AR2||Zin,A+(1/j2πfC2)×(1+R6R5)],

where *Z*_in,*A*_ is the input impedance of operational amplifiers used in the circuit (same operational amplifier used for both stages) and *f* is the working frequency. In addition, according to Figure 2(a), the input impedance of the neural amplifier is

(13) $$\begin{array}{c} Z_{\text{in}}\approx \frac{1}{j2\pi fC_{1}}+R_{1}||Z_{\text{in},A}. \end{array}$$

With the electronic components used for the amplifier, (12) and (13) can further be simplified. Two low-noise CMOS amplifiers (LMP7702, Texas Instruments, Dallas, Texas, USA) [66–71] were used to build both the first and second stages of the neural amplifier. This particular operational amplifier has excellent low-noise characteristics. Its input referred noise reduces from its highest point of 120 nV/*√*Hz at 1 Hz to 9 nV/*√*Hz at 1 KHz and maintains this low noise level for frequencies above 1 KHz. The LMP7702 also has small input capacitance *C*_in_ ≈ 25 pF, which is two orders of magnitude smaller than *C*_1_ = 1.5 nF. Thus, *A*_1_ ≈ 1 and the imaginary part of *Z*_in,*A*_ becomes the dominant term in (13) Therefore, for the frequency range (100 Hz to 5 kHz) of interest in extracellular neural recording, (12) and (13) are simplified to

(14) $$\begin{array}{c} A_{0}\left(f\right)\approx 1\cdot \left[\frac{R_{2}}{R_{2}+\left(1/j2\pi fC_{2}\right)}\left(1+\frac{R_{6}}{R_{5}}\right)\right], \end{array}$$

(15) $$\begin{array}{c} Z_{\text{in}}\approx \frac{1}{j2\pi fC_{\text{in}}}. \end{array}$$

There are several points worth mentioning in the design. The RC high-pass filter (*C*1 and *R*1) is used to reject the DC offset induced by the liquid junction potential and half-cell potential in the glass micropipette with a 3-db cut-off frequency at 0.1 Hz. Another RC high-pass filter (*C*2 and *R*2) sandwiched between the first unity-gain stage and the second gain stage is designed to reject other low frequency interference from the environment with a 3-db cut-off frequency at 80 Hz. A 1 V offset was added as a reference to the amplifier output voltage in order to capture both the positive and negative sides of the extracellular action potentials.

### 2.3. Intrinsic Noise and Input Capacitance Measurements

To measure the intrinsic noise of our neural amplifier, the input of the amplifier is directly shorted to the input ground as depicted by Figure 2(b). A data acquisition system (NI USB-6341, National Instruments, Austin, Texas, USA) was used to record the output voltage at a sampling rate of 50 kS/s. A Fourier transform is performed on the measured output voltage and subjects the voltage spectrum to a 100 to 5000 Hz digital filter to reject unwanted noise outside the signal bandwidth. The data processing and analysis were performed using Origin (OriginLab, Northampton, USA) data processing software. The intrinsic amplifier noise was also simulated by Multisim software (Austin, Texas, USA).

In addition, the input impedance of the amplifier is also measured because it greatly influences the SNR as described by (11) and (15). According to (15), the input impedance of the amplifier can be approximated by *C*_in_ within the frequency range of measurements. The input capacitance of the amplifier consists of the intrinsic input capacitance of the operational amplifier and the parasitic capacitance of the printed circuit broad. In order to measure the total input capacitance of the amplifier, the frequency response of the amplifier is measured as depicted by Figure 2(c). A high quality D/A converter (RP 2.1, Tucker-Davis Technology, Alachua, USA) was used to generate a sinusoidal voltage with a 1 V peak-to-peak amplitude for frequencies from 1 Hz to 2000 Hz. The sinusoidal voltage was connected in series to a 10 MΩ resistor and the input stage of the neural amplifier. Since the first-stage operational amplifier has a unit gain with a 2.5 MHz bandwidth, the 3 dB cutoff in the frequency spectrum is solely due to the RC low-pass filter formed by the 10 MΩ resistor and the overall input capacitance. Therefore, the overall input capacitance can be calculated using the time constant relationship for this measured 3-dB bandwidth (*f*_*c*_):

(16) $$\begin{array}{c} C_{\text{in}}=\frac{1}{2\pi \left(10\;\text{M}Ω\right)f_{c}}. \end{array}$$

### 2.4. Micropipette and Environmental Noise Estimation Using Dead Brain Tissue

To estimate the noise of the amplifier together with the micropipette electrode and the influence of environmental (power-line) interference, dead brain tissue was used for easy accessibility. The setup for measuring the noise of these conditions is shown in Figure 2(d). A 70-day-old wild type Mongolian gerbil (*Meriones unguiculatus*) was sacrificed. All experimental procedures involving animals were approved by the University of Colorado's institutional animal care and use committee (IACUC, protocol number B-88412(05)1C). Once deep anesthesia was confirmed, the gerbil's head was removed and put into 0.9% normal saline, and the brain was excised. Subsequently, the brain was washed in 0.9% normal saline to remove the blood from the surface, put into a 25 mL glass beaker filled with saline, and maintained at 37 degrees Celsius. A single barrel glass micropipette (GC150F-10 borosilicate glass, Harvard Apparatus, Edenbridge, United Kingdom) was pulled to a 1-2 *μ*m tip diameter using a DMZ-Universal puller (Zeitz Instruments, Martinsried, Germany). The glass pipette was filled with 27% saturated sodium chloride solution using a carbon fiber needle (Microfil MF 28G67-5, World Precision Instrument, Sarasota, USA) such that the complete electrode had a total impedance of 5 MΩ to 15 MΩ. Subsequently, the filled micropipette was inspected with a microscope to make sure the tip was not broken, and no air bubbles were left in the tip of the micropipette. Then the pipette was positioned in an electrode holder attached to a piezo-electric drive (Inchworm controller 8200, EXFO Burleigh Products, Victor, NY), and a silver-silver chloride wire was inserted into the solution in the pipette. The input of the neural amplifier was connected to the other end of the wire. Meanwhile, a copper wire having a 1 mm diameter was immersed into normal saline as a ground for the neural amplifier. Then the pipette was slowly advanced into the brain tissue and the output of the neural amplifier both in an electrically quiet (light off) and an electrically noisy environment (light on) was measured. The electrical noise was introduced through a 50 W halogen light bulb placed 10 cm away from the brain. The output voltage of the micropipette neural amplifier was recorded by the NI USB-6341 data acquisition system for further data analysis.

### 2.5. *In Vivo* Recordings from the Brain of an Anesthetized Rodent

**Figure 3:**
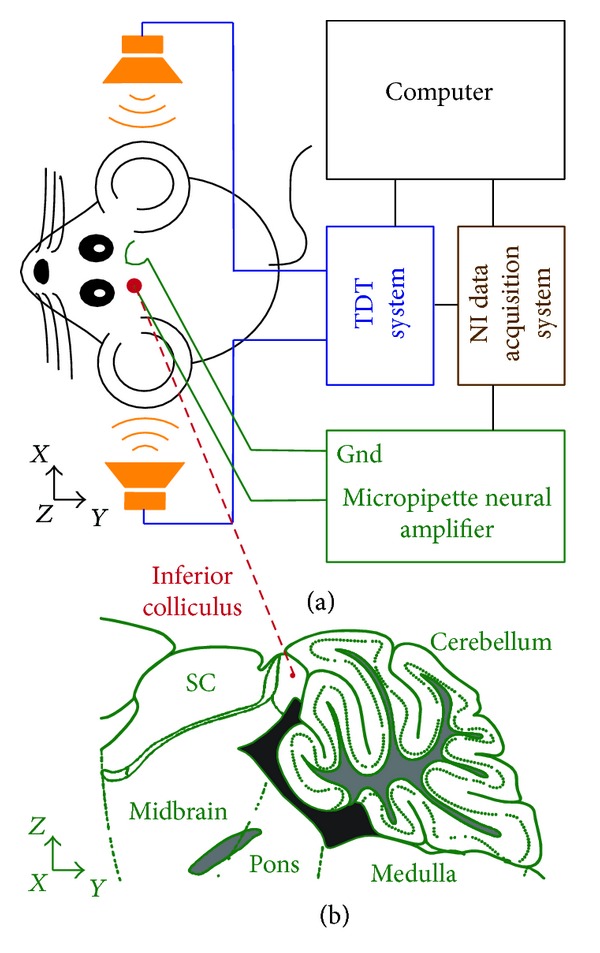
(a) Experimental setup of measuring neural voltage responses in the inferior colliculus (IC) induced by auditory stimulation in the ears of an anesthetized gerbil. The auditory stimulation is triggered by a high-quality signal generator. A sharp-tipped micropipette is inserted into the brain of the gerbil held by a piezo drive to reach to the IC for recording. The neural voltage is amplified by the micropipette neural amplifier. The output voltage of the amplifier is subsequently digitized by an analog-to-digital converter and recorded by the computer for further analysis. (b) Illustrated diagram of a gerbil's brain showing the relative position of the IC.

In this section, animal testing was applied to test the efficacy of the micropipette neural amplifier in* in vivo* neural recording as depicted by Figure 3. All experimental procedures using animals were approved under University of Colorado IACUC protocol number B-88412(05)1C. During these experiments, a glass micropipette was advanced into an auditory area, the inferior colliculus (IC) of a Mongolian gerbil. We recorded action potentials from neurons in the IC in response to sound stimulation of the animal's ears.

#### 2.5.1. Animal Preparation  (See  [72])

Before surgery, a 68-day-old gerbil was anesthetized by initial intraperitoneal injection (0.5 mL/100 g body weight) of a mixture of Ketamine (20%) and Xylazine (2%), both diluted in physiological saline. During surgery and the recording session, a supplemental dose of 0.25 mL/100 g body weight of the same mixture was administered subcutaneously every 30 minutes. Constant body temperature was maintained using a thermostatically controlled heating pad.

Skin and tissue overlying the top part of the skull were removed and a small silver hook was attached to the subcutaneous tissue near the head as the ground of the amplifier. Custom-made earphone holders were attached to the head, allowing for the safe insertion of earphones into the ear canal. The animal was then transferred to a sound-attenuated chamber and mounted in a custom made stereotaxic instrument. The animal's position in the stereotaxic apparatus was standardized with reference to stereotaxic landmarks on the skull. For electrode penetrations of the IC, a small hole of approximately 1 mm^2^ was cut into the skull lateral to the lambdoid suture. Micromanipulators were used to position the recording electrode according to the landmarks on the skull surface and a reference point. The meninges overlaying the cortex were removed and normal saline was applied to the opening to prevent dehydration of the brain. After successful recordings were taken, the animal was sacrificed by injection of an overdose of ketamine and xylazine.

#### 2.5.2. Setup for Neural Recording Triggered by Sound Stimulation of Inferior Colliculus Neurons

When the animal was positioned in the stereotaxic instrument, a single barrel glass micropipette as described above was positioned above the opening in the skull and advanced perpendicular to the skull surface using a piezo drive which could be remotely controlled from outside the sound-attenuated chamber. A real-time processor with a high-quality analog I/O (RP 2.1, Tucker-Davis Technology, Alachua, FL, USA) was used to generate modulated sinusoidal wave signals to drive the two headphones. The modulation of the sinusoidal wave was programmed with a RPvdsEx program (Tucker-Davis Technology, Alachua, FL, USA), which was used to control the real-time processor. The sinusoidal wave was modulated to have a 20 ms rise time, a 20 ms fall time, and a 50 ms/550 ms ON/OFF period. Two programmable attenuators (PA 5,TDT) were used to attenuate the sinusoidal wave before it was applied to two speaker drivers (ED1, TDT) to drive the headphones. The micropipette neural amplifier was applied to amplify the neural action potential signals. The NI USB-6341 data acquisition system was used to record the output signal of the micropipette neural amplifier.

## 3. Results

### 3.1. Characterization of the Neural Amplifier

#### 3.1.1. Intrinsic Noise of the Neural Amplifier

**Figure 4:**
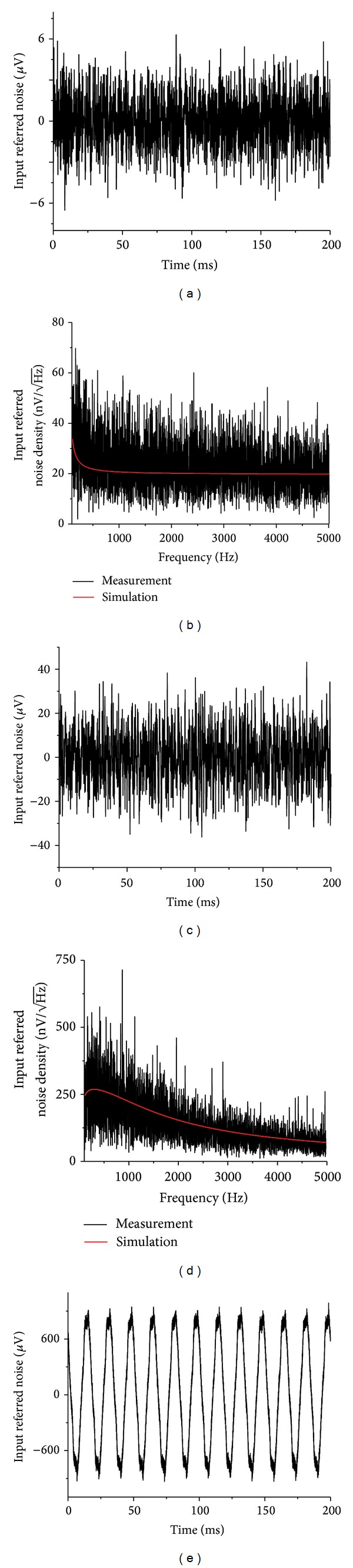
(a) The measured intrinsic input referred noise of the neural amplifier. (b) Circuit simulation and measurement results of the intrinsic input referred spectral noise density of the neural amplifier calculated from the data in (a). (c) Input referred noise of the neural amplifier with the glass micropipette electrode measured in dead gerbil brain tissue with an electrical quiet environment (light off) (d) Circuit simulation and measurement results of the input referred spectral noise density calculated from the data measured in (c). (e) Input referred noise measured in dead gerbil brain tissue in an electrical noisy environment (light on).

Here we report the measurement results obtained by using the techniques described in the method section.  Figure 4(a) shows the time trace of the measured input referred noise of the micropipette neural amplifier for a time period of 200 ms. The measured noise voltage is 11.95 *μ*Vpp in peak-to-peak voltage or 1.81 *μ*V_rms_ in root-mean-square voltage [73–75].  Figure 4(b) shows the corresponding simulated (red) and measured noise densities of the intrinsic amplifier. Within the frequency range of interest for neural recording (100 to 5000 Hz), the amplifier noise is simulated to be 9.50 *μ*Vpp or 1.44 *μ*V_rms_ using Multisim. The measured noise density is higher than the simulated results and we attributed this discrepancy to imperfections and manufacturing deviations when making the printed circuit broad and electronic components. Based on our circuit simulation, the amplifier suffers from flicker noise which linearly decays from 100 Hz to 500 Hz. At 100 Hz, the noise density is highest of 32 nV/*√*Hz with our simulated result. The noise density reduces to 20 nV/*√*Hz above 500 Hz where thermal noise dominates.

#### 3.1.2. Input Capacitance Measurement and SNR Estimation

The input capacitance of the micropipette neural amplifier is measured in accordance with the methodologies described in the previous section. As shown in (11), the input capacitance of the amplifier is very important in determining the signal-to-noise ratio of the amplifier. The frequency response of the amplifier is measured (data not shown) and the 3-dB cut-off frequency of the amplifier was determined to be 751 Hz. Thus, the input capacitance is estimated to be *C*_in_ = 21.23 pF using (16). The measured input impedance conforms to our analysis stated in (13) and is dominated by the input capacitance of the CMOS amplifier LMP7702 (25 pF as stated in the datasheet). Thus, if a glass micropipette with 5 MΩ impedance is used, the SNR of a 500 *μ*Vpp 1 KHz sinusoidal signal is calculated to be 8.74 according to (11).

### 3.2. Noise Characterization of the Neural Amplifier with Micropipette Using Dead Brain Tissue

#### 3.2.1. Noise of the Amplifier with Glass Micropipette

Noise measurement to characterize the performance of the amplifier can be performed using dead brain tissue extracted from a gerbil. In order to measure the background noise generated by the amplifier and the glass micropipette, we insert the micropipette directly into the dead brain tissue submerged in a saline solution to maintain tissue freshness.  Figure 4(c) shows the measured input referred noise of the micropipette amplifier over a course of 200 ms.  Figure 4(d) shows the corresponding noise densities for the simulated (red) and measured results. To avoid any environmental interference impairing our result, the brain tissue and the amplifier were enclosed by a Faraday cage. The input referred noise was measured at 78.54 *μ*Vpp, or 11.9 *μ*V_rms_. For the glass micropipette with a 5 MΩ impedance, the 3-db cut-off frequency is ~1.5 KHz. Thus, the thermal noise generated by the glass micropipette is calculated to be 73.59 *μ*Vpp or 11.15 *μ*V_rms_ and the overall input referred noise is 74.18 *μ*Vpp or 11.24 *μ*V_rms_ using (9), which is in agreement with the measurements.

#### 3.2.2. Environmental (Power-Line) Noise

Environmental (power-line) noise can also be estimated using dead brain tissue.  Figure 4(e) shows the noise acquired when a power line placed near our measurement setup powering a 50 W halogen light-bulb. Power-line noise overwhelms the intrinsic noise of the amplifier and the micropipette, and the overall input referred noise was measured to be ~1.75 mVpp. Compared to a typical neural spike of 50 *μ*Vpp to 500 *μ*Vpp, the power-line noise is extremely strong. For this reason, a Faraday cage and other environmental noise elimination strategies are necessary for* in vivo* extracellular neural recording of conscious animals.

### 3.3. *In Vivo* Measurements of Neural Responses to Sound Stimulation from an Anesthetized Gerbil

**Figure 5:**
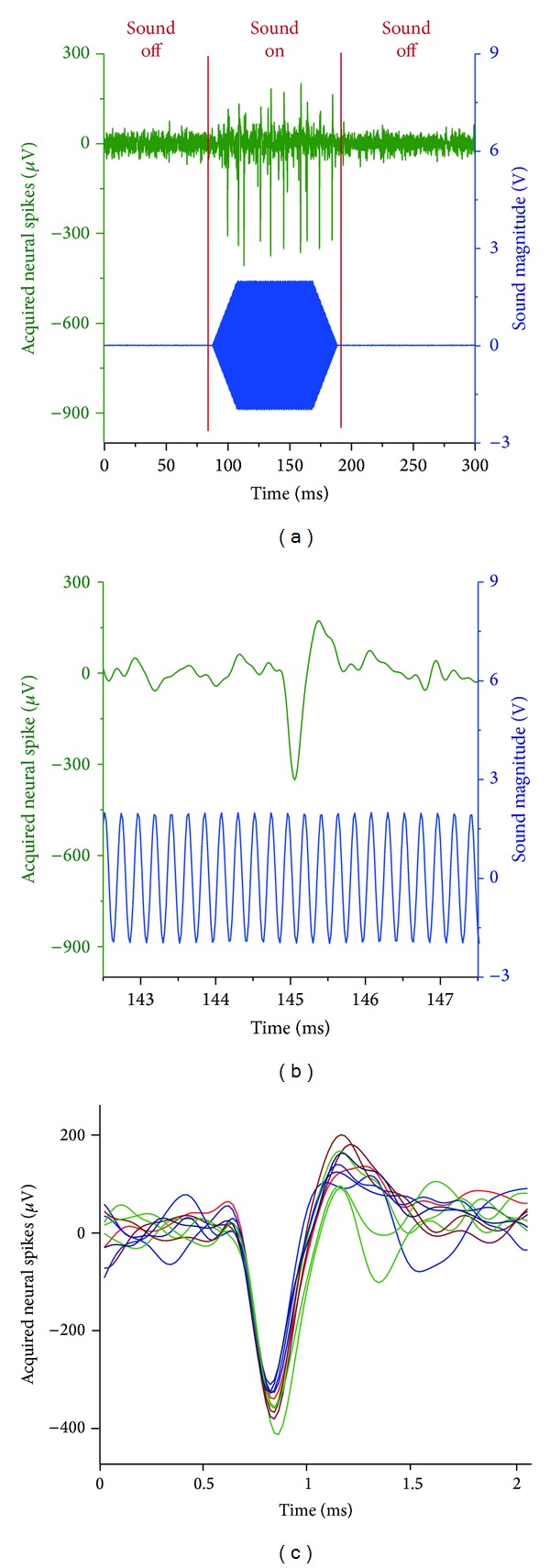
(a) Extracellular neural voltage spiking (green) acquired by the micropipette neural amplifier in the inferior colliculus (IC) of an anesthetized gerbil. The ears of the gerbil are attached to audio speakers and sound frequency sweeps are periodically played to the anesthetized gerbil. The recorded neural voltage spiking was recorded simultaneously with the sound frequency sweeps during the experiment. The magnitude of the sound sweep (blue) is plotted together with the neural voltage spiking (green) in the same graph to show that the neural spiking is only observed with the auditory stimulated. (b) The neural voltage (green) and the sound magnitude (green) of (a) are magnified in time for a period of 5 ms to show better details of both signals. (c) Individual neural voltages of (a) are plotted on top of one another. All individual neural signals have a similar temporal shape which indicates that the signals all originated from a single neuron.

The IC is an auditory neural target and neural voltages are generated when triggered by sound.  Figure 5(a) shows neural spiking recorded by the micropipette neural amplifier when the anesthetized gerbil was triggered externally by a sound sweep.  Figure 5(b) shows the magnified neural spike and the corresponding sound magnitude over a 5 ms time period for better resolution of the signals.

The occurrence of the neural spikes strongly correlates with the sound excitation and the neural spikes only appeared when external sound sweep was active. The neural signal likely originated from a single neuron in the IC, since it disappeared when the micropipette was retracted by several *μ*m. The signal was restored when the micropipette was moved back to its original location.  Figure 5(c) overlays the neural spikes of Figure 5(b) on top of each other to demonstrate that all the measured action potentials have similar temporal shapes, indicating that all the measured neural responses are indeed originating from a single neuron [76]. These results demonstrate the efficacy of the micropipette amplifier for recording extracellular neural signals originating from a single neuron for* in vivo* experiments. The average voltage of the background noise was measured at around 100 *μ*Vpp or 15.15 *μ*V_rms_ (averaged over the first 50 ms of the measurement when no sound was played). This background level is close to the measured noise level with dead gerbil brain (78.54 *μ*Vpp, or 11.9 *μ*V_rms_). The neural spikes have an average maximum voltage of ~500 *μ*Vpp. Thus, the SNR of the micropipette neural amplifier is estimated to be larger than 5.

We tabulated the noise performance of the micropipette amplifier in Table 1. The simulated results of the intrinsic amplifier noise (*V*_*A*_), the micropipette thermal noise (*V*_*Re*_), and the overall amplifier noise (*V*_total_) were compared to the empirical results of the intrinsic amplifier noise (*V*_*A*_) and the overall noise (*V*_total_) measured in a dead gerbil brain and in the brain of an anesthetized gerbil. (The micropipette thermal noise cannot be directly measured empirically since other noise sources, such as environmental noise, cannot be completely excluded in the laboratory.) The comparison shows that the noise characteristics of our micropipette amplifiers are closely matched to the theoretical predictions. The measured overall noise (*V*_total_) only differs from the theoretical prediction by 6% and 35% when measured in a dead gerbil brain and in an anesthetized gerbil.

## 4. Discussion

In this paper, we have developed an equivalent circuit and a noise analysis model for extracellular neural recordings using a low-noise amplifier with a glass micropipette. The models were developed to help understand and to quantify the various noise sources contributing to the overall noise of the recording. Experimental measurements were also proposed to verify the accuracy of our models. In addition, we have designed and constructed a low-noise two-stage amplifier based on the mathematical models. The intrinsic noise of the amplifier and environmental (power-line) interference were measured using a dead gerbil brain. In addition, we also performed* in vivo* neural recordings from the brain of an anesthetized gerbil to confirm that the amplifier has adequate SNR for extracellular* in vivo* neural recordings. The recorded neural spiking at the IC, which is an auditory nucleus in the brain, correlates with the sound stimulation in the animal's ear.

The main finding of our study is that two chief noise sources degrade the neural voltage signal in the recordings, namely, the intrinsic noise (*V*_*A*_^2^) of the amplifier and the thermal noise of the glass pipette (4*kTR*_*e*_), as shown in (11). Since intrinsic noise is one of the major noise contributors, it is important for circuit designers to develop noise-reduction strategies in the design of amplifiers with low noise characteristics. Many research groups have been working vigorously on improving amplifier designs, and the input referred noise of most neural amplifiers described in literature is already better than 5 *μ*V_rms_ [8–18, 77]. Some designs achieve an overall noise as low as 1-2 *μ*V_rms_ [8, 11, 77]. Besides intrinsic amplifier noise, our noise analysis also indicates that the thermal noise generated by the electrolyte inside the micropipette is another significant noise contributor to the overall measurement noise. Millar and Barnett [13] reported a noise figure of 13 *μ*V_rms_ over a bandwidth of 100 Hz to 8 kHz for a 1 MΩ glass micropipette. Budai [8] also reports a 5.6 *μ*V_rms_ thermal noise with a bandwidth of 5 kHz for 0.4 MΩ electrode. Therefore, it is important to develop strategies in future designs to lower the resistance of the glass pipette to further reduce thermal noise.

In this paper, we used excised gerbil brain tissue to characterize environmental interference with neural recordings. Our measurements conclude that interference from power lines is significant and can easily exceed neural signals from extracellular recordings. Therefore, it is necessary to enclose the entire neural recording setup with a Faraday cage as a shield from these noise sources. Faraday cages, however, are bulky and also add another layer of difficulty to neuroscience experiments, in particular for* in vivo* neural recordings from conscious behaving animals where the experimental setup requires a large area. Therefore, it is imperative to develop noise-cancelation strategies on the amplifier circuit itself to eliminate the need for Faraday cages for noise shielding.

We have also successfully recorded neural responses triggered by external auditory stimulation. Our recorded spike trains from neurons in the IC are in step with the delivery of the sound frequency sweep. In modern neuroscience research, the ability to measure neural signals from a single cell and to correlate the recordings with behavioral responses, such as sound sweeps, is important in the study of brain functionality, such as studies of sensory processing, memory and decision making, as well as understanding the pathophysiology of neural disorders, such as Parkinson's diseases. Therefore, engineering designs that allow an investigator to advance the electrode accurately into a specific brain nucleus and the ability to control neural activity* in vivo* in conscious animals are critical for many future neuroscience studies.

Another important development is the use of optogenetic proteins to excite or inhibit action potentials using optical stimulation in the brain [78]. Optogenetics is a new frontier in neuroscience research and uses light-gated ion-channels that allow stimulation and inhibition of a specific cell-type of a brain nucleus using biochemical techniques and optical methods [79, 80]. Optical stimulation can be delivered to a brain nucleus via optical fibers or implanted LEDs. It is therefore imperative to develop electrical systems that allows simultaneously stimulating and/or inhibiting neural responses and records neural voltages at the same or another remote neural target in the brain. Some optogenetic applications, however, require the use of high-current LED drivers which can generate a strong electrical interference to the sensitive recording amplifiers. Optical interference from these high-power optical sources to the recording amplifier has been reported [80]. Therefore, we plan to include the strong electrical interference generated by these high-current LED drivers in our noise models to guide our future designs of electronic systems for neural manipulation.

## 5. Conclusion

In this paper, we proposed a comprehensive mathematical model to predict the overall noise of a neural amplifier using a glass micropipette as the recording electrode in extracellular neural recording. Our model shows that both thermal noise generated inside the glass micropipette and the intrinsic amplifier noise are major contributors to the overall noise of the amplifier. We compared the results calculated from our proposed noise model to experimental measurements obtained from a dead gerbil brain, as well as* in vivo* measurements from an anesthetized gerbil. We experimentally measured noise spectral densities and the measurements were well matched to our theoretical predictions, validating the accuracy of our proposed model. Moreover, neural response originating from a single neuron measured in the IC was observed to be temporally correlated with auditory stimulation with a conscious gerbil.

## Figures and Tables

**Table 1:** Comparison of noise levels of theoretical predictions and experimentally measured results. Values in parenthesis indicate the percentage increase in the experimental results over the simulated results.

	Theoretical predication (*μV*_rms_)	Measurements in a dead gerbil brain (*μV*_rms_)	*In vivo* measurements with an anesthetized gerbil (*μV*_rms_)
Intrinsic amplifier noise (*V*_*A*_)	1.44	1.81 (26%)	1.81 (26%)
Micropipette thermal noise (*V*_*Re*_)	11.15	—	—
Overall noise (*V*_total_)	11.24	11.90 (6%)	15.15 (35%)
